# Stress-induced glucocorticoid receptor activation determines functional recovery following ischemic stroke

**DOI:** 10.1186/2040-7378-2-18

**Published:** 2010-09-22

**Authors:** Fabiola CR Zucchi, Norah-Faye Matthies, Noora Badr, Gerlinde A Metz

**Affiliations:** 1 Canadian Centre for Behavioural Neuroscience, Department of Neuroscience, University of Lethbridge, 4401 University Drive, Lethbridge, Alberta, T1K 3M4, Canada

## Abstract

**Background:**

A major consequence of stroke is permanent motor disturbance, such as postural imbalance and loss of skilled movement. The degree of neuronal and functional loss and subsequent recovery after stroke is influenced by hypothalamic-pituitary-adrenal axis activation and the response to glucocorticoid hormones. This study investigated if recovery after stroke is related to glucocorticoid receptor (GR) activation in a rat model of stroke.

**Methods:**

Adult male rats were pre-trained and tested in a skilled reaching task and received a focal ischemic motor cortex lesion. One group of animals received daily restraint stress starting one week pre-lesion up to three weeks post-lesion. Immuno-histochemical analysis of GR expression was performed to determine receptor activation.

**Results:**

Stress reduced reaching success in naïve animals and diminished recovery of limb use. Exaggerated functional loss in stressed rats was related to increased GR activation in the lesion hemisphere as indicated by nuclear GR location.

**Conclusion:**

These findings provide a mechanistic link between stress-induced motor disability and GR activation in a rat model of stroke. The elevated receptor activation proposes synergistic effects of stress and stroke to modulate the impact of glucocorticoids on motor system function at the genomic level. The modulation of GR biosynthesis may alter responsiveness to stroke treatment and compromise recovery.

## Introduction

Common symptoms of stroke include motor disturbances, such as postural imbalance and disturbed skilled movement [1-3]. The degree of spontaneous functional recovery after stroke is determined by inflammatory processes, which are modulated by stress and activity of the hypothalamic-pituitary-adrenal (HPA) axis [4].

Stress and high levels of glucocorticoids (GCs) are associated with poor stroke outcome and high morbidity [5-8]. The experience of psychological distress is associated with increased stroke risk and represents a predictor of fatal stroke [9-13]. Stress has been recognized as a critical variable in determining the success of stroke therapy and rehabilitative interventions [14,15]. Because adverse experience modulates neuronal plasticity [16,17] the intrinsic properties of the HPA axis, such as GR density, may explain the large variability in recovery rates among individual patients [18]. Because GC therapy also represents a potential treatment for stroke, investigation of glucocorticoid receptor (GR) regulation is critical to explore potential therapeutic avenues.

This study describes the effects of psychological stress on GR expression and activation, and its impact on recovery and outcome after ischemic lesion in a rat model. The findings suggest that stress-induced regulation of GR expression after ischemic stroke may influence HPA axis feedback mechanisms.

## Materials and methods

### Animals

Twenty-two adult male Long-Evans rats (400-500 g) raised at the University of Lethbridge vivarium were used. For participation in skilled reaching, animals were food restricted to maintain 90-95% of baseline body weight. Animals were matched to two groups based on baseline reaching success: *Stress *(n = 11), and handled *Controls *(n = 11). All procedures were performed in accordance with the guidelines of the Canadian Council for Animal Care and approved by the local Animal Welfare Committee.

### Motor cortex lesion

Rats were anesthetized using isoflurane in an oxygen/nitrous oxide mixture (isoflurane 4% for initiation, 2% for maintenance at an oxygen flow rate of 2.0 l/min). Motor cortex devascularization was induced contralateral to the paw preferred in skilled reaching [19]. Briefly, the skin over the skull was incised and the skull was exposed. Using a fine dental burr, a craniotomy was performed at the following coordinates: -1.0 to 4.0 mm anterior-posterior, 1.5 to 4.5 mm lateral to Bregma. The dura and blood vessels were carefully wiped off using a sterile cotton tip. The skin was sutured and the rats were given analgesic (Temgesic, Schering-Plough, Brussels, Belgium). Rats were allowed to recover in individual cages on a heating pad until fully awake and were then returned to their home cages.

### Stress paradigm

The stress regimen was performed 7 days prior, and 21 days after motor cortex lesion. Rats were placed individually in a transparent Plexiglas cylinder [20-22]. The cylinder (5 cm inner diameter) had perforated ends to allow for ventilation and maintained the animals in a standing position without compression of the body. Restraint stress was given starting at 9:00 AM. Animals were restrained for 20 minutes and were tested 10 minutes later in the skilled reaching task [23,21,25]. Furthermore, on the last day of baseline (pre-stress) and after 3 weeks of post-lesion behavioural tests, blood samples were collected after a 10 minute post-stress interval. Thus, both behavioural testing and blood sampling took place at a time when elevated corticosterone levels after restraint stress can be expected [21,23].

### Skilled reaching task

**Figure 1:**
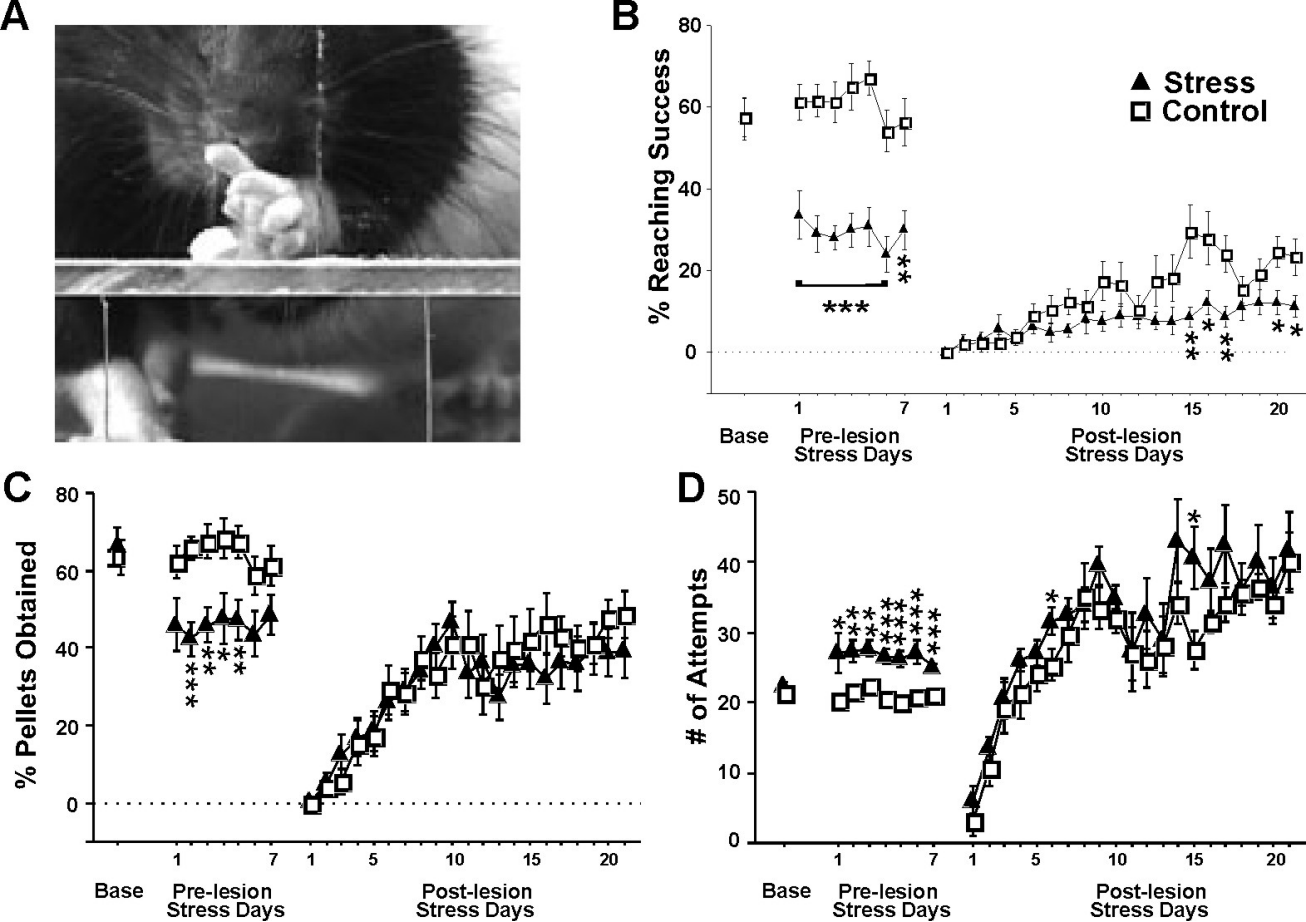
**Stress induces motor impairments**. (A), photograph of a rat reaching for a food pellet. (B), success percent; (C), pellets obtained percent; (D), number of attempts to grasp 20 pellets. All data are presented as group means + SEM. * p < 0.05, ** p < 0.01, *** p < 0.001, compared to the *Control *group.

The rats were trained in the single pellet reaching task to assess skilled forelimb function [26,27] (Figure 1A). The reaching boxes were made of clear Plexiglas (40 cm × 45 cm × 13 cm). The front wall of the box had a 1.3 cm wide vertical slit, allowing the rats to reach for a food pellet located on a shelf attached to the outside of the box. The shelf was located 4 cm above the floor. On top of the shelf were two indentations (5 mm in diameter, and 1.5 mm deep), each aligned with one side of the slit. These indentations stabilized the pellet and were located 1.5 cm away from the front wall [27]. In each training session, rats were placed individually in the reaching box and a food pellet (45 mg each, BioServ, Frenchtown, NJ) was placed contralaterally to the rats' preferred reaching paw. To readjust their body position, rats were trained to walk to the rear end of the box before reaching for a new pellet. Each rat was given 20 pellets per training and test session.

A successful reach was defined as obtaining the pellet on the first attempt, withdrawing the paw through the slit and releasing the pellet to the mouth. Success was calculated using the following formula:

$$\text{Percentage of reaching success }=\frac{\text{number of successful reaches}}{\text{20}}\times \text{100}$$

The percentage of total number of pellets obtained was measured by counting the number of pellets eaten, regardless of whether the pellet was grasped and eaten on the first attempt. If the rat dropped the pellet, it did not count as a pellet eaten. The percentage of total pellets obtained was measured using the following formula:

$$\text{Percentage of total pellets obtained}=\frac{\text{number of pellets obtained}}{\text{20}}\times \text{100}$$

To assess reaching accuracy, the number of attempts to grasp a single pellet was averaged.

Once reaching success rates during training sessions reached an asymptotic level, performance was recorded for 5 days of baseline testing. These values were averaged for further analysis (pre-stress). After the start of the stress regimen animals were tested in skilled reaching for 7 days prior and for 21 days after lesion.

### Blood samples

Blood samples were taken from the tail vein on the last day of baseline (pre-stress), and three weeks post-lesion (the day before sacrifice). An average of 0.6 ml of blood was collected 10 minute post-stress interval under 4% isoflurane anesthesia. Blood samples were collected between the hours of 9:10-10:30 AM. No behavioural testing was performed on days on which blood samples were taken. Plasma corticosterone concentrations were determined by radioimmunoassay using commercial kits (Coat-A-Count, Diagnostic Products Corp., Los Angeles, CA) [28].

### Histology and immunohistochemistry

After completion of behavioural tests, rats were sacrificed with an overdose of pentobarbital (Euthansol; CDMV Inc. Québec, Canada) and transcardially perfused with phosphate buffered saline followed by 4% formaldehyde. Brains were cut at 10 μm thickness. Forty-five sections per brain were cut in each of the anterior lesion site, the middle section and the posterior lesion site. Three sections of each area were used for immuno-histochemical analysis.

#### Infarct volume

Cresyl Violet stained brain sections (10 μm) were used to analyze infarct volume. Cross-sectional volumes of both hemispheres were calculated using a Zeiss Axiovision 4.3 microscope (Zeiss, Jena, Germany). Infarct volume was measured according to the Cavalieri method [29]. The following formulas were used (VH: volume of a hemisphere; VTL: volume of tissue lost):

$$\begin{array}{l} \text{VH}=\text{ average }\left(\text{average area of a complete coronal hemisphere }–\text{ ventricles }–\text{ area of damage}\right)\\ \;\times \text{ interval between sections }\times \text{ number of sections} \end{array}$$

VTL= tissue remaining in normal hemisphere – tissue remaining in injured hemisphere

#### Immunostaining

Serial coronal sections were incubated overnight in primary antibody rabbit anti-GR (Santa Cruz Biotech; 1:50). Controls were performed omitting the primary antibody. Sections were incubated with a secondary anti-rabbit biotinylated antibody (Vectastain ABC Kit: peroxidase rabbit IgG, 1:200), which was detected using the Elite ABC-peroxidase kit (Vectastain ABC Kit, Vector) with DAB as chromogen (according to [30,31] but modified). Sections were examined under light microscopy.

#### Quantitative analysis of GR expression and activation

**Figure 2:**
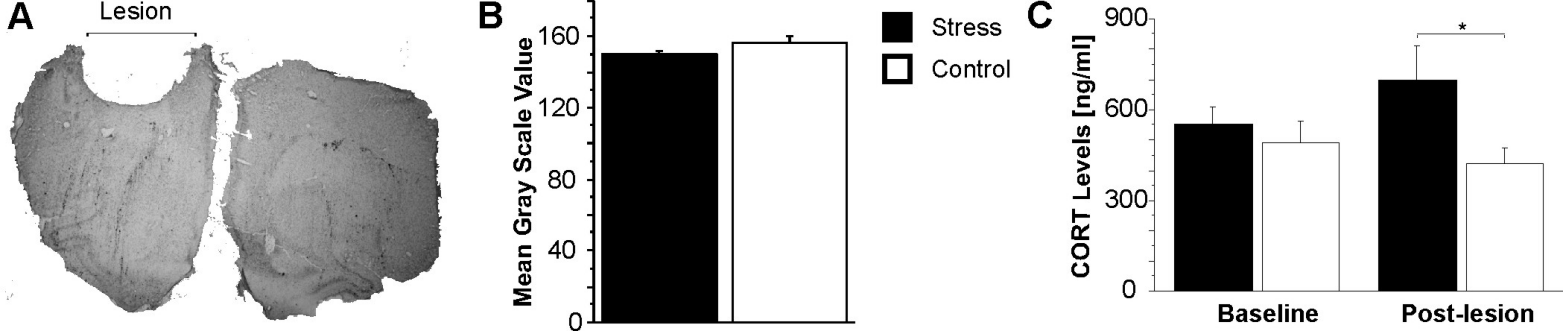
**Chronic stress activates the stress response in absence of GR density alteration**. (A), Coronal brain section showing GR immune-histochemistry; (B), total GR quantification; (C), plasma corticosterone levels (CORT). All data are presented as group means ± SEM. * p < 0.05, compared to the *Control *group.

**Figure 3:**
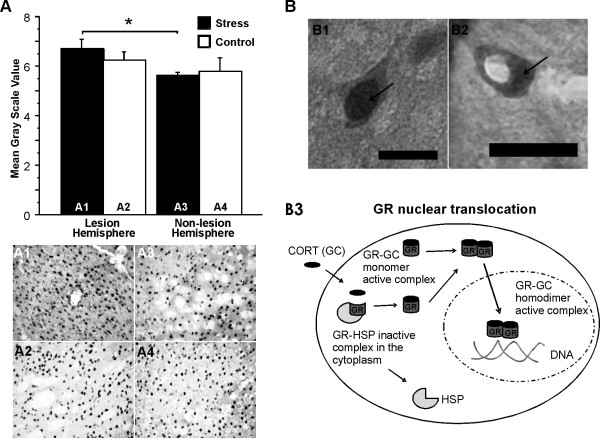
**Stress modulates GR activation after ischemic lesion**. (A), GR nuclear density; (B), GR nuclear translocation. Lesion increased activated GR density in the lesion hemisphere compared to the non-lesion hemisphere in *stress *(A1 and A3) and *control *(A2 and A4) groups. Stress increased GR activation in the lesion hemisphere (A1) when compared to the non-lesion hemisphere (A3) (*p < 0.05). GR is constitutively expressed in the cytoplasm (arrow in B2), and inactive as a GR-HSP complex. GC enters the cell and binds to a GR dissociating the GR-HSP complex (Schema B3). Consequently, GR is activated when a GR-GC dimer is formed and translocated into the nucleus (arrow in B1; schema B3). Scale bars: 10 μm.

Photographs were captured digitally and panoramic pictures (1× lens) from coronal brain sections were taken (Figure 2A). Three motor cortex areas in the lesion and non-lesion hemispheres were photographed (40×; Figure 3A1-A4). GR expression was measured using ImageJ software (NIH, Bethesda, Maryland, USA). Color images were converted to monochromatic photo images to determine mean gray scale values.

GR activation was quantified by selecting the cellular stained nucleus (according to [32-34] but modified). Cellular location of GR was analyzed semi-quantitatively (100× lens; Figure 3B1, B2).

### Statistical analysis

Statview software version 5.0 (SAS Institute, 1998) was used to perform analyses of variance (ANOVA), unpaired student t-tests for between-group comparisons and paired t-tests for within-group comparisons in the single pellet reaching task, and for GR expression patterns across hemispheres. A p-value of less than 0.05 was chosen as significance level. All data are presented as mean ± standard error of the mean.

## Results

### Skilled reaching

#### Reaching success

There was a significant difference in the success rate between groups (F(1,28) = 56.34, p < 0.001). *Stress *animals had lower reaching success than *Control *animals (pre-lesion stress: day 1, t(21) = -3.80, p < 0.001; day 2, t(21) = -5.58, p < 0.001; day 3, t(21) = -5.58, p < 0.001; day 4, t(21) = -4.84, p < 0.001; day 5, t(21) = -5.72, p < 0.001; day 6, t(21) = -4.45, p < 0.001; day 7, t(21) = -3.48, p < 0.01; post-lesion stress: day 15, t(21) = -2.91, p < 0.01; day 16, t(21) = -2.14, p < 0.05; day 17, t(21) = -2.94, p < 0.01; day 20, t(21) = -2.63, p < 0.05; day 21, t(21) = -2.28, p < 0.05; Figure 1B). There was a decline from baseline to post-lesion testing in *Stress *animals, and from pre-lesion to post-lesion in *Control *animals (*Stress*: t(10) = 10.22, p < 0.001, t(10) = 5.14, p < 0.001; *Control*: t(11) = 10.77, p < 0.001).

#### Number of pellets obtained

There was significant difference in the number of pellets obtained between groups (F(1,28) = 21.71, p < 0.001). The *Stress *group showed a decrease on days 2, 3, 4 and 5 pre-lesion when compared to *Controls *[day 2: t(21) = -4.36, p < 0.001; day 3: t(21) = -3.48, p < 0.01; day 4: t(21) = -2.52, p < 0.05; day 5: t(21) = -3.02, p < 0.01; (Figure 1C)]. Performance decreased from baseline to pre-lesion in *Stress *animals, and from pre-lesion to post-lesion in *Controls *(*Stress*: t(10) = 4.56, p < 0.001; *Control*: t(11) = 5.93, p < 0.001).

#### Number of Attempts

Overall there was a significant group difference in the number of attempts to grasp a food pellet (F(1,28) = 17.14, p < 0.001). *Stress *animals made more attempts than *Controls *on all pre-lesion days and days 6 and 15 post-lesion (pre-lesion: day 1, t(21) = 2.42, p < 0.05; day 2, t(21) = 3.59, p < 0.01; day 3, t(21) = 3.57, p < 0.01; day 4, t(21) = 3.61, p < 0.001; day 5, t(21) = 5.56, p < 0.001; day 6, t(21) = 3.87, p < 0.001; day 7, t(21) = 5.83, p < 0.001; post-lesion: day 6, t(21) = 2.08, p < 0.05; day 15, t(21) = 2.57, p < 0.05; Figure 1D). *Controls *made more attempts after lesion than pre-lesion (t(11) = -5.27, p < 0.001). *Stress *animals made more attempts pre-lesion than at baseline (t(10) = -4.92, p < 0.001).

### Infarct size

The lesion included the primary and secondary motor cortex as well as the forelimb and hind limb areas of somatosensory cortex (Figure 2A). There was no significant difference in infarct size between groups. The *Stress *group lost on average 18.02 mm^3 ^of tissue and the *Control *group lost on average 12.14 mm^3 ^of tissue. There was no correlation between infarct size and skilled reaching success (r = 0.37).

### Plasma corticosterone

The *Stress *group had higher plasma corticosterone (CORT) levels compared to *Controls *post-lesion (t(21) = 2.81, p < 0.05; Figure 2C).

### Glucocorticoid receptor expression

*Stress *animals showed slightly lower total GR expression than *Controls *(Figure 2B). There was higher GR activation in the lesion hemisphere in *Stress *animals when compared to the non-lesion hemisphere (t(7) = 3.43, p < 0.05; Figure 3A1-A3). There was slightly higher GR activation in the lesion hemisphere of *Stress *animals, and lower GR activation in the non-lesion hemisphere, when compared to *Control *animals (Figure 3A).

Semi-quantitative analysis allowed the discrimination between nuclear (Figure 3B1) and cytoplasm (Figure 3B2) GR sub-location. GR nuclear density was increased near the lesion site.

## Discussion

The present findings provide a mechanistic link between stress-induced motor disability and biochemical changes in a rat model of stroke. At the behavioural level, stress diminished skilled limb use in naïve rats and hindered motor recovery from an ischemic infarct thus confirming previous studies [35,21,24,25,37]. Furthermore, chronic stress increased circulating corticosterone concentrations. At the molecular level, chronic restraint stress modulated GR activation of central motor systems that cause permanent alterations in GC susceptibility. These findings support the notion that stress drives biochemical changes that are accompanied by lasting functional loss.

The main finding of the present study revealed an additive effect of stress and stroke to enhance GR activation in the lesion hemisphere. While enhanced GR activation occurred without concomitant changes in infarct size, diminished recovery in these animals suggests that elevated GC levels are detrimental to functional outcome. Stroke can induce an inflammatory response in the brain, what might affect the immune-endocrine communication, such as GC levels, causing the alteration of essential biological functions. Although corticosteroids may represent a potential treatment for ischemic cerebral edema [38], even physiologically elevated GC levels may modulate edema formation and recovery after ischemic infarct [24,36]. The latter studies suggest that chronic stress and associated immuno-endocrine interactions may promote disease aggravation. These observations are not surprising given the large body of evidence documenting central GC effects. Elevated GC levels may promote pro-inflammatory cell migration, cytokine production, and transcription factor activity in the brain [4] resulting in necrotic cell death [39].

High GC levels render neurons more susceptible to neurological insults via disruption of anti-apoptotic factor [40] and neurotrophic factor expression [15,41,42]. GC-mediated regulation of these factors might exaggerate infarct size [43], limit structural plasticity and the capacity to compensate for functional loss [24,37].

In human acute stroke the GC component of the stress response can be harmful, at least when cortisol reaches high blood concentrations within the first days after stroke to exaggerate ischemic injury and neuronal death [12]. Furthermore, corticosteroid treatment has been shown to be ineffective for some survivors from acute ischemic stroke [44]. The concert of anti- versus pro-inflammatory effects of GCs complicates the interpretation of clinical trials.

The mechanism of action of GCs is through its binding with the type I mineralocorticoid receptor (MR) and the type II GR [45]. MRs are mostly occupied by GCs at basal levels [46]. GRs have low affinity for GCs and become occupied at the time of stress-induced GC elevation. GRs typically reside in the cytoplasm of the cell, bound to chaperone heat shock proteins until GCs enter the cell. Upon ligand binding the GR-GC complex is released from the chaperone complex to translocate to the cell nucleus (see Figure 3B3). Once in the nucleus, the GR-GC complex binds to the DNA, influencing transcription [47,48]. These changes may be directly linked to lasting alterations in brain function and stroke outcome.

Enhanced GR activation in the lesion hemisphere suggests altered sensitivity of the HPA axis induced by stress. GRs are involved with reactive feedback to restore disturbed homeostasis [49]. Modulatory influences of GR, such as GC negative feedback, may dampen the HPA response [50]. Once GCs induce neuronal loss, low GR density could reduce the HPA axis negative feedback, thus promoting further GC production [51,52].

The balance of MR- and GR-mediated effects exerted by GCs is critical for homeostatic control [53]. Here we found increased GR activation in the lesion hemisphere, suggesting effective negative HPA feedback regulation that confines deleterious effects of stress to protect or promote neuronal survival. This notion is supported by considerable motor recovery in the absence of exaggerated infarct size after stress.

Additional to central regulation of the stress response, GRs are critical for physiological sustainability. GRs modulate synaptic plasticity associated with the plasmatic membrane acting through second messengers to regulate signal transduction cascades [54]. Increased GR expression in the lesion hemisphere could therefore promote plasticity and maintain integrity of existing pathways thus facilitating compensation after stroke.

Since GC therapy represents a treatment option for clinical stroke to contain inflammatory processes, the investigation of the role of stress and lesion in GR regulation is critical to explore future therapeutic avenues. The current findings suggest that one possible mechanism to affect stroke outcome is brain GR modulation, and consequent alteration of stress responsiveness and GC therapy. The investigation of GR regulation therefore is a critical step towards designing effective therapies and rehabilitation strategies for stroke survivors.

## Competing interests

The authors declare that they have no competing interests.

## Authors' contributions

FCRZ: Experimental design, behavioural testing, immuno-histochemical procedures, data analysis and interpretation, statistical analysis, manuscript preparation. N-FM: Brain microtomy, immuno-histochemical procedures, assistance with data acquisition. NB: Brain microtomy, immuno-histochemical procedures, assistance with data acquisition. GAM: Experimental design, project supervision, data interpretation, manuscript preparation. All authors read and approved the final manuscript.
